# Railway Spine

**Published:** 1894-09

**Authors:** 


### EDITORIAL DEPARTMENT.

F. E. DANIEL, M. D., Editor.
S. E. HUDSON, M. D., Managing Editor.
A. J. SMITH. M. D., Galveston, Associate Editor.

EDITORIAL STAFF:

PROF. J. E. THOMPSON, M. D., Texas Medical College, Galveston; Surgery.
PROF. WM. KEILLER, M. D., Texas Medical College, Galveston; Obstetrics and Gynecology.
PROF. DAVID CERNA, M. D., Texas Medical College, Galveston; Therapeutics.
PROF. A. J. SMITH, M. D., Texas Medical College, Galveston; Medicine
DR. ISADORE DYER, Tulane University; Dermatology.
DR. R. H. L. BIBB, Saltillo, Mexico; Foreign Correspondent.
DR. ROBT. MORRIS, Charity Hospital, N. O.; Clinical Reports

### "RAILWAY SPINE."

Whether the suggestions of a recent editorial article in the JOURNAL have or have not been absolutely correct in indicating the destruction of belief in the so-called "railway spine" as the *causa vivendi* of the American Association of Railway Surgeons, to an observer desirous of maintaining an impartial attitude, the idea presents enough plausibility to allow of its consideration—the more so when one has had an opportunity of attending one of the sessions of this organization. One would hesitate to entertain such an idea for even a moment, were it not for the relative position of matters; a large body of the paid medical representatives of corporations liable to serious pecuniary losses, because of the recognition of such an affection as that known as "railway spine," practically unanimous, as far as their utterances in convention are concerned, in the repudiation of the disease and the explanation of its phenomena by suggestions protective of their employers and ungenerous to the injured claimants. Such a combination is, to say the least, quite as unsatisfactory as the rapid recovery of a litigant patient after the payment of his claim, and is even less simply explicable. Under the circumstances brought about by the character of remarks made by Mr. Clark Bell, of New York, and Dr. W. B. Outten, of St. Louis, at the recent Galveston convention of the railway surgeons, in which the former, a prominent railway attorney, characterizes the affection as "a vampire," as "the Nemesis of modern surgery," and as "invented as a means of procuring enormous verdicts from railway corporations in accident cases"; and the latter, a prominent railway surgeon, blames the very existence of the affection upon the neurologists who recognize it—under these circumstances the editorial writer may feel justified in plainly asserting his suspicions. Nor can these now meet with hasty and angry denial without proof; such a repudiation is in no wise scientifically justified and is liable only to aggravate the prime suspicion.

With the exception of the august body of gentlemen named, and an inconsiderable number of scattered individuals not now known to the writer as non-interested in their position, the actual existence of the affection known as "railway spine" is denied by none. Men not trained not to know it have little difficulty in recognizing it, and differ not as to its existence, but as to its variations, its causation, its pathogenesis and pathology, its prognosis and its cure—differences wide enough, it is true, to indicate the ignorance of the profession as to its nature, but not wide enough to persuade any one of its non-existence. Were it not for the clearness of these expressions mentioned, and their evident purpose, it would be grossly unfair to credit these gentlemen with the desire of creating the belief that railway spine is a grand piece of deception, with no other purpose than legal robbery, and that it is but an invention and without actual basis; but their own words open them to such a conclusion. There are few of the warmest advocates of "railway spine" but will readily acknowledge that in individual cases suggestion may be found as an important causative factor; but quite as few scientific observers, who have paid any attention to the affection, seek to deny that there is under this tendency to develop suggested symptoms an actual pathological basis, either physical or psychical, in some way connected with injury from trauma or psychical shock, or both. Strümpel (*Schmidt's Jahrb.*, Bd. 242, No. 5), in his remarks before the German Congress of Internal Medicine, held in April, 1893, says with truth: "If one were to suspect the reality of the disease because of the variability of certain symptoms, and because of the tendency of the patient to apparent exaggeration, we neurologists would have to acknowledge nearly half of our private patients as frauds."

One should not be misled by the multitude of names attached to the disease to believe that this diversity indicates an absence of real foundation, and that they, therefore, mean nothing at all —more nearly the truth would be the sum of their meanings chronic concussion of the spine, railway spine, railway brain, traumatic neurosis, traumatic hysteria, traumatic neurasthenia, traumatic psychoneurosis, *et al.* The simple fact that none of these names have been proved to rest upon a positively established pathology is without significance to the point in question, and these many names only indicate the cloudiness and obscurity of our knowledge of the nature of the affection of whose existence we have reason to believe. So the nations have had many names for God, of whose attributes little is known, but of whose existence there are ample evidences to those who will see. No one contends that "railway spine," as a name, is anything but an arbitrary term, used in connection with an affection often of much wider import than a spinal injury due to a railway accident. "Railway spine" may occur from very diverse forms of injury, all sorts of mechanical shock other than railway shock, as well as the latter, lightning stroke, sunstroke, and purely psychical shock; and there are doubtless, too, in many cases predisposing causes of significance if one searches closely for them. (It seems to the writer of much more profit, scientifically, that these gentlemen should occupy their energies in seeking the influences which heredity, alcoholism, syphilis and similar possibly predisposing conditions may exert upon the development of traumatic hysteria or neurasthenia following a railway shock—and of more profit, too, to their employers—than that they should, in the face of the facts the profession holds in regard to the affection, deny its existence and characterize it as an invention of a clever man.) It is by no means limited to its spinal manifestations. It may present no symptoms at all referable to the spine or cord, may be purely cerebral or peripheral in its symptoms, or may show such general distribution in its symptomatology that one can not refuse to accept it as a disease of the general nervous system. So too its phenomena are as variable and as peculiar to the case as their distribution; purely subjective, as pains and tenderness, paræsthesias, hyperæsthesias or anæsthesias of the general and, too, of the special senses; relatively objective symptoms, as the contraction of the visual field, tremors, cardiac ataxic phenomena, with various nutritive and vasomotoric symptoms, associated with neurasthenic evidences and psychical manifestations of a hysteric or hypochondriac order. It is an easy assertion on the part of those who desire to deny the reality of the affection that so wide a symptom complex is subversive of the idea of a definite pathology, and is best explained by a voluntary or unwitting deception. Rather may it be assumed that this diversity of distribution and character of symptoms is expressive of general nervous implication, rarely complete in individual cases but possible in any. Most writers of the last few years are willing to acknowledge only the rare occurrence of willful deception; but freely accord as an expression of the low psychical tone of the patient, the influence of suggestion and the tendency on the part of the patient to exaggeration of existing symptoms. Almost throughout the discussion of the question before the German Congress already referred to, partaken in by such notable German physicians as Strümpel, Wernicke, Hitzig, Bruns, Baümler, Hoffmann, Jolly, Ziemssen, Schultze, Unverricht and others, the same idea prevails. Jolly distinctly stated his belief that whatever of tendency to simulation exists is part of the disease; that the tendency to exaggeration in the ordinary cases of hysteria has long been recognized, but never used as an argument by the profession or others that these patients are not sick. Unverricht alone declared that he regarded deception as entering to any great degree into these cases, and this gentleman advised the use of chloroform narcosis as a means of unmasking any simulation —by his very proposition acknowledging that there are real cases from which to separate the unreal ones. Hitzig and Strümpel both protested against this idea of permitting the affection to rest on its objective phenomena alone, in that because of its, in their opinion, essentially psychical nature, even in the absence of the objective symptoms, one may not entirely deny the presence of the disease. It may be stated as a fair expression of the ideas developed in this discussion that there is no doubt of the reality of such an affection, and that whatever differences were expressed as to the frequence of the disease, as to how far it may be and sometimes is simulated willfully or unwittingly, only serve to accentuate the general acceptance of its actuality.

And in cases where in many accidents (as in Dr. Outten's records contrasting the number of cases of railway spine met among railway passengers and railway employees, and in the records of Byron Bramwell—*Brit. Med. Journal*, November 18, 1893—as to the absence of railway spine among the colliers of England) few instances of the affection are recognized, it is by no means to be accepted without proof that they do not exist. Bruns (*Schmidt's Jahrb.*, *ibid*,) in abstracting the remarks of Bramwell, adds with force: "The only question is whether the

English colliers do not actually have traumatic neuroses, or whether they only say nothing of it since there is no use of speaking." Moreover contrasted with the many positive cases reported by men whose acumen is otherwise undoubted, and whose ability to detect fraud may be reasonably held as high as that of their opponents, these negative evidences weigh but little. Further the testimony of well developed cases appearing without litigation, and therefore without any stimulation of the factor of deception or exaggeration is of far more value than the records of negation advanced by the gentlemen named. Reference here may well be made to the eighteen cases reported by Potts (*University Med. Magazine*, p. 777, 1893), in which the symptoms presented were almost uniformly subjective, and therefore open to the suspicion of simulation, but in which the utter absence of chance for litigation almost entirely removes this suspicion. The writer may also with propriety refer here to a case occurring within his own experience of a traumatic neurasthenic following a railway injury, in which the psychical symptoms proceeded so far that when his case was practically won, and the railway attorney had signified his readiness to allow the claim, he became positively insane, refused the money (which was quite ample) took his case out of the hands of his successful lawyer, and disappeared for a long time. His subsequent reappearance, re-employment of his former lawyer, and acceptance of the offered money, do not weaken the fact that this man's impaired psychical condition was positively unbalanced for a time at least by the excitement of gaining his desired claim—a circumstance most unlikely in case of deception.

It would seem quite as profitable, because in many cases of insanity objective symptoms are not prominent or absent, and because the picture of the disease is almost, if not entirely, made up of subjective symptoms, to deny the existence of the insanity; or because insanity may be mimicked to declare it a vampire and invented to obtain a free living at the hands of the public.

Nor yet are all the symptoms of the traumatic-neurosis subjective and simulable; there are in reality many which, recognized by a reasonably careful physician, defy simulation. One must hold it as impossible that a patient should repeatedly be able by simulation to produce the irregularly contracted fields of vision when examined upon the commonly used perimeters. One must hold it as impossible that a patient could produce the ataxia-like irregularities of the heart, or the irregularly occurring tachycardias so often met with in these cases, or the irregular areas of sweating that are occasionally met with, or the occasional flushings and pallors that appear, as well as the occasional very apparent anomalies of temperature in different parts of the body surface. There are, too, various trophic changes which are quite unlikely to be simulated, the changing of the color of the hair to gray in a few months or weeks, as has occurred in a case under the writer's care, a development of a coarseness of the hair in a short space of time, changes in the skin, causing the complexion to take on the muddy appearance so often seen among the insane, especially the paretic insane, or in the rapid development of scruffiness and dryness in parts shortly before normal in appearance. So, too, tremors ought to be easily detected, when simulated; recourse to Unverricht's method, as mentioned above, should surely determine the reality of persistent tremors. It is unfortunate, but it is no argument against the reality of the disease, that the basis of these and, too, of the subjective symptoms, can not be with certainty named. This must await the solution of time, by which most problems of human interest are usually dissipated. It seems not unlikely that the pathology may be varied, and the distinctions already being made in the different cases may be widened. More chances for anatomical study are needed, although they may be as unproductive as they have been in many cases of insanity. It seems credible that, as in insanity, a group of cases have been found depending upon a widespread and minute sclerotic process, we may eventually accept a similar pathology, as indicated by the work of Friedmann, (*Munich Medical Wochensch*, XLI-20, 21, 22, 1893), and others, in a group of these cases; or may even be able to detect a finer molecular change in the nervous elements of one locality or the entire system, the result of nervous shock by trauma or psychical injury, as indicated by Strümpel in the above-mentioned discussion, as well as by many others of the earlier contentions as to the nature of the affection.

At any rate, whatever the pathology as eventually found out, to whatever degree the patient himself is responsible for the severity or multitude of his symptoms, whatever of his affection is imposed upon him by others by suggestion, the matter is now above accusations of "invention" and declarations of complicity on the part of men trained to know nervous phenomena, when they meet them, and generally far enough separated from any pecuniary reason for taking other than a scientific interest in the cases brought to their notice.

